# The N. Y. Court of Appeals on Adulteration

**Published:** 1889-12

**Authors:** 


### THE N. Y. COURT OF APPEALS ON ADULTERATION.

In a case of adulteration recently before the Court of Appeals of New York, the court said :

"It is notorious that the adulterations of food products have grown to proportions so enormous as to menace the health and safety of the people. Experience has taught the lesson that repressive measures which depend for their efficiency upon proof of the dealer's knowledge and of his intent to deceive and defraud, are of little use and rarely accomplish their purpose. Such an emergency may justify legislation which throws upon the seller the entire responsibility of the purity and soundness of what he sells, and compels him to know and to be certain. Therefore, the Commissioner says that want of knowledge on the part of the seller is no defense."

